# Ischemia induced repolarization dispersion changes and ventricular arrhythmia: Validation of Frank vectorcardiography parameters: A review

**DOI:** 10.1016/j.hroo.2025.02.005

**Published:** 2025-02-17

**Authors:** Lennart Bergfeldt, Lennart Gransberg, Gunilla Lundahl

**Affiliations:** Department of Molecular and Clinical Medicine, Institute of Medicine, Sahlgrenska Academy, University of Gothenburg, Gothenburg, Sweden

**Keywords:** Myocardial ischemia, Dispersion, Arrhythmogenesis, Arrhythmia suppression, Vectorcardiography, Spinal cord stimulation, Pig model

## Abstract

Changes in dispersion of ventricular repolarization is a salient electrophysiological feature of acute myocardial ischemia and linked to life-threatening arrhythmias. In 9 studies, we applied Frank vectorcardiography not only for noninvasive monitoring of cardiac electrophysiology during acute ischemia induced during percutaneous coronary interventions, during clinical myocardial infarction, and in an occlusion-reperfusion pig model, but also for prognostic purposes after coronary events. Myocardial ischemia was in humans and pigs associated with significant changes in the ST segment, Tamplitude, Tarea, ventricular gradient, and T vector loop morphology. In the pig model, a link between dispersion changes and the occurrence and suppression (by spinal cord stimulation) of ventricular arrhythmias was found. Prognostically, after recent acute coronary events, the spatial peak and mean QRS-T angles were superior. In summary, vectorcardiography-based parameters were validated as reflecting changes in repolarization dispersion during myocardial ischemia in humans and pigs, and in pigs linked to the occurrence and suppression of ventricular arrhythmias.

**Key Findings**

▪ Changes in vectorcardiography (VCG)-based dispersion parameters reflected ischemia-induced action potential heterogeneities during coronary occlusion/myocardial infarction, similarly in humans and pigs.
▪ Accentuated VCG-based dispersion parameters and increased heart rate preceded VF in pigs, and spinal cord stimulation reduced the magnitude of VR dispersion and sustained and nonsustained ventricular tachycardia but not VF.
▪ After acute coronary syndromes, increased peak and mean QRS-T angles added predictive value regarding subsequent cardiac death including sudden cardiac death on top of 9 demographic and clinical risk factors, while VCG parameters reflecting changes in dispersion during acute ischemia did not.
▪ The T vector loop was more abnormal (more circular and bulgier) in patients with amplitude signs of left ventricular hypertrophy on electrocardiography and became more abnormal during acute myocardial ischemia (ie, T vector loop morphology changes have pathophysiological correlates).

## Introduction

Sudden cardiac death (SCD) is a major health problem, supposedly mainly due to ventricular arrhythmias, and coronary artery disease is the most common underlying etiology in adults above 35 to 40 years of age.^1^, ^2^, ^3^ Although mortality and morbidity related to acute coronary syndromes have decreased when patients receive optimized guideline-recommended hospital care and secondary prevention, prehospital mortality remains significant.^4^ Primary prevention therefore remains crucial and requires mechanistic insights.

While ventricular extrasystoles are triggers, a substrate is required for the initiation and sustenance of a ventricular arrhythmia.^2^ Ventricular electrical dispersion (heterogeneity)—a key element in arrhythmogenesis in myocardial ischemia—is in general caused by regional differences in ventricular activation sequence, gap junction distribution, and the duration and morphology of action potentials.^5^, ^6^, ^7^, ^8^, ^9^ Knowledge about ventricular dispersion and its changes in general and in coronary artery disease in particular are therefore of both theoretical and clinical importance.

Dispersion of cardiac electrical activity is a prerequisite for the electrocardiogram (ECG) (ie, dispersion of ventricular depolarization for the QRS complex and of ventricular repolarization [VR] for the T wave). In that sense, it is a physiological phenomenon, but changes in dispersion might create a pathophysiological substrate for arrhythmias. Changes in VR dispersion have attracted more attention than changes in depolarization dispersion.^10^ Because dispersion of VR is a prerequisite for the T-wave, different methods have been applied to deal with its quantitative aspects.^11^ One method, QT dispersion or the interlead difference in the QT interval on the 12-lead ECG, was proposed in the early 1990s and initially received a great deal of interest but was abandoned after approximately a decade for both theoretical and methodological reasons.^12^ During this period, Badilini and colleagues in 1995 (subsequently corroborated by Kors and colleagues)^13^^,^^14^ showed that QT dispersion reflected the T vector loop morphology. In their discussion, Badilini and colleagues raised the question whether vectorcardiography (VCG) would be a suitable methodology for dispersion analysis. A crucial issue related to this suggestion is whether VCG-based measures of dispersion reflect experimentally proven changes in action potential heterogeneities such as those occurring during acute myocardial ischemia. Providing an account on this matter from the results of 9 studies emphasizing their common context is the main topic of this report. It is based on data and results from studies initiated 1995 and onward and published between 2000 and 2016 applying Frank VCG in a similar way in humans and pigs to ensure uniform methodology.

Thus, inspired by Badilini and colleagues,^13^ we first asked ourselves how acute ischemia affected T vector loop morphology, QT dispersion, and Tarea^.^ Coronary occlusion during angioplasty (ie, percutaneous coronary intervention [PCI]) offers a human experimental model of myocardial ischemia allowing serial observations and was first used. In subsequent studies, including the acute phase of myocardial infarction, other candidate VR dispersion parameters were explored in humans and pigs and in the latter model also the relation between changes in VR dispersion and the occurrence of ventricular arrhythmia and attempt to its suppression. Finally, prognostic information provided by VCG-based measures was included.

**Figure 1:**
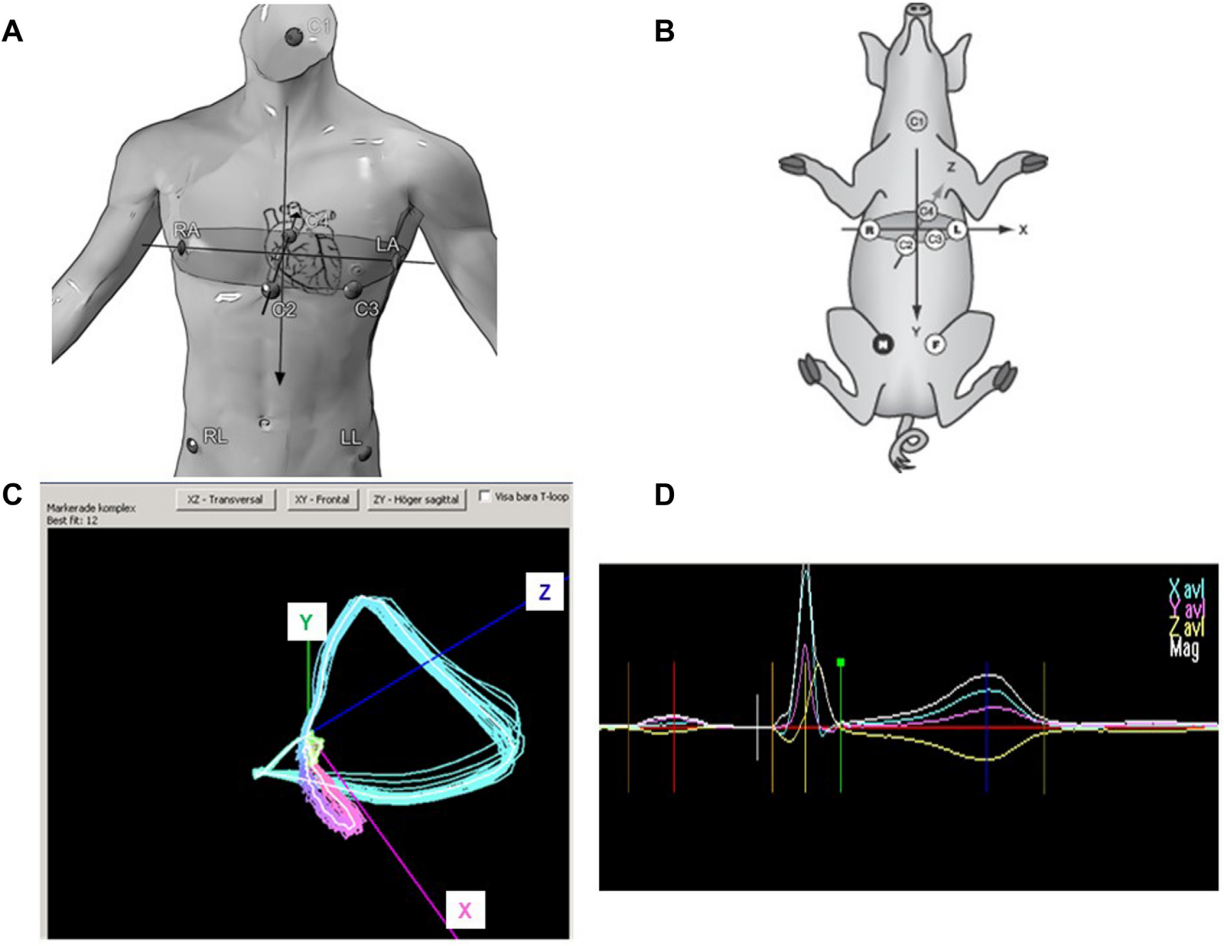
Vectorcardiography according to Frank. Electrode positions in humans (A) and in a pig model (B). C: A 3-dimensional recording of P, QRS, and T vector loops with the white loops marking the means of the sample from 1 individual. XZ is the transversal, XY is the frontal, and YZ is the right sagittal plane. D: The PQRST complexes in the X, Y, and Z directions in separate colors and the calculated mean global PQRST complex in white (denoted Mag for magnitude). Panel A is reproduced with permission from Wecke L. Cardiac memory studies in two human models. PhD thesis, Karolinska Institutet, Stockholm, Sweden; 2006. Panel B was reproduced with permission from Odenstedt and colleagues (Elsevier license 5880220229265).^27^ Panel C was adapted with permission from Bergfeldt and colleagues^21^ and reused under a Creative Commons license (CCC 4.0).

**Figure 2:**
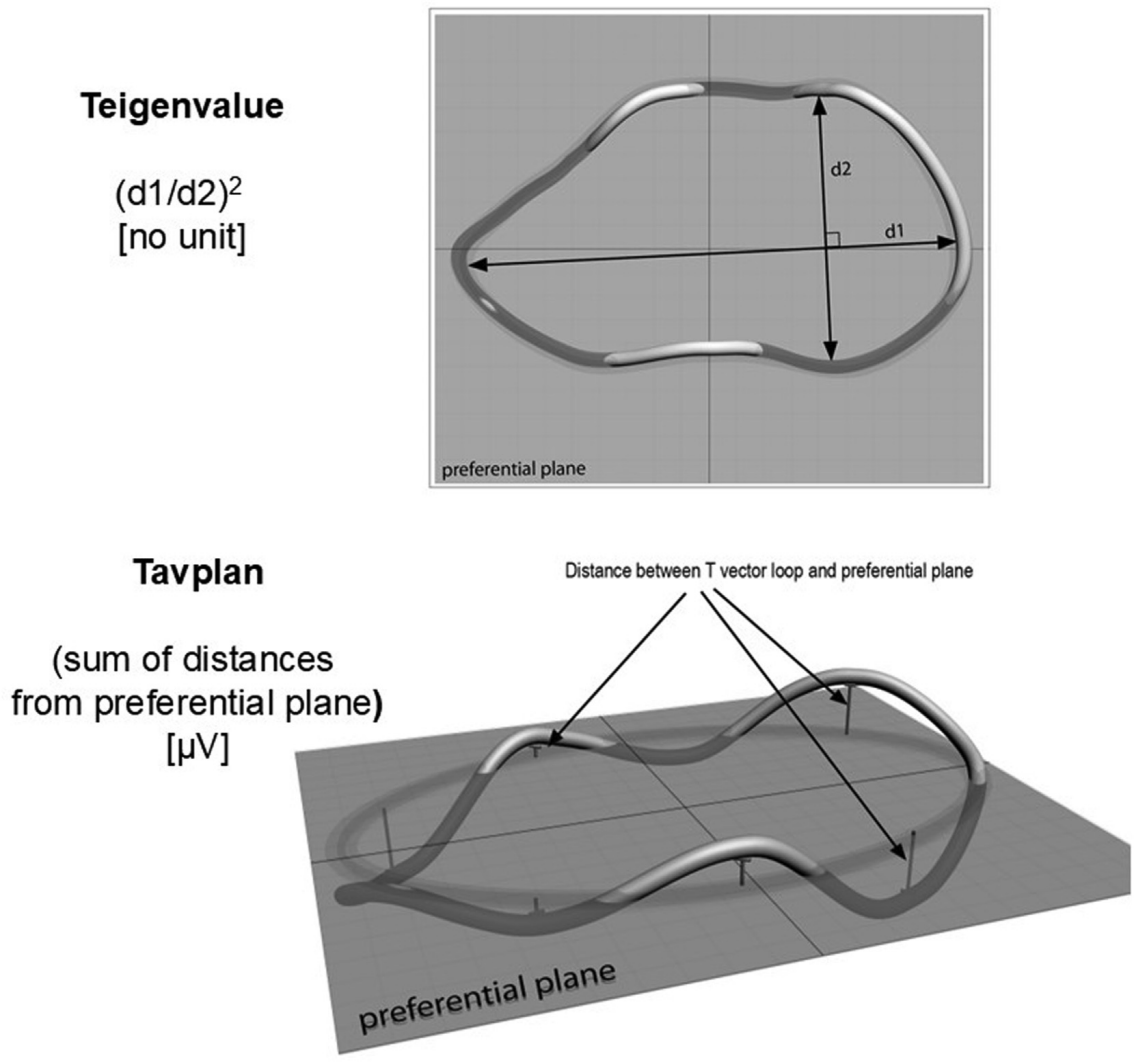
T vector loop morphology descriptors. The T vector loop is in one preferential plane that differs between individuals. (Top) The loop’s roundness with the 2 longest perpendicular axes marked. The loop is normally elliptical, and for an ellipse the eigenvalues are proportional to the squares of the 2 largest axes. Teigenvalue is therefore calculated as (d_1_/d_2_)^2^ and is unitless (in the literature sometimes referred to as ℷ_1_ and ℷ_2_). The higher the value is, the more elongated the ellipse is. For the perfect circle, Teigenvalue is 1, which is the most abnormal. (Bottom) The bulginess of the loop calculated as the mean value of absolute distances of deviations above and below the preferential plane as Tavplan in μV. Reproduced with permission from Wecke L. Cardiac memory studies in two human models. PhD thesis, Karolinska Institutet, Stockholm, Sweden; 2006.

**Figure 3:**
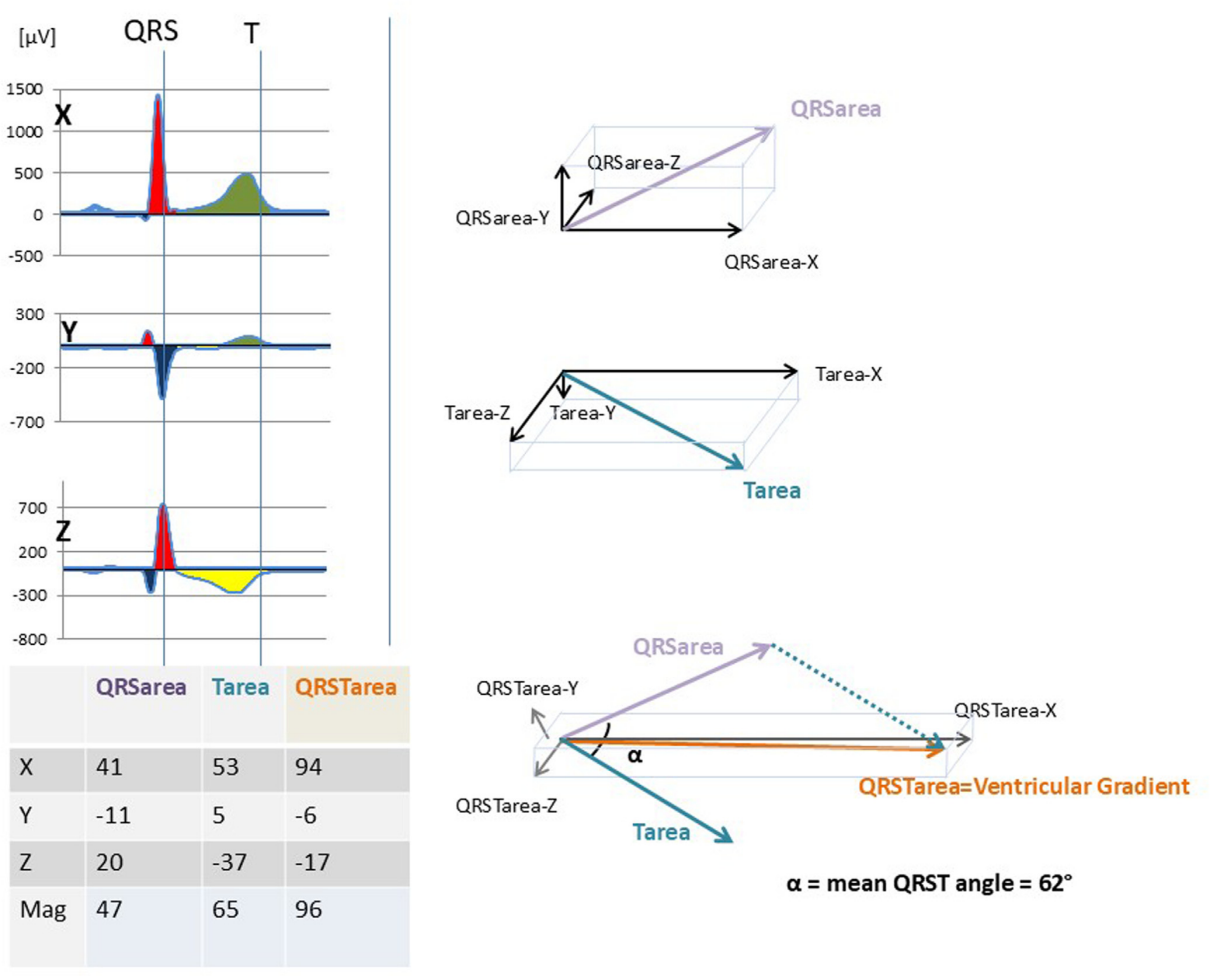
QRS_area_ and Tarea vectors and their relation to the mean QRS-T angle and the ventricular gradient. The QRS_area_ and Tarea are calculated from the root mean square areas in X, Y, and Z directions. The ventricular gradient (ie, QRST integral and spatial ventricular gradient, in μV) is the sum of the QRS_area_ and Tarea vectors and thus dependent on both their magnitude and direction. The mean QRS-T angle is the angle (°) between these vectors and only dependent on their direction. Thus, there is a complex inverse relationship between these two parameters. Reproduced with permission from Bergfeldt and colleagues^21^ and reused under a Creative Commons license (CCC 4.0).

VCG is based on recordings in a system in which the vector loops are formed by calculated orthogonal X, Y, and Z leads (Figures 1A–1C).^15^ VCG parameters are calculated from the vector loops and from a 3-dimensional (ie, global or magnitude) PQRST complex formed by the X, Y, and Z leads (Figure 1D). In addition to conventional ECG intervals obtained from the global PQRST complex, several other parameters can be defined and calculated such as vector loop characteristics, the peak and mean QRS-T angles, and global dispersion measures (eg, Tamplitude, Tarea, and the ventricular gradient [VG]) (Figures 2 and 3).^16^, ^17^, ^18^, ^19^, ^20^, ^21^ One aim of this report was therefore to address the internal validity of VCG as a tool for noninvasive assessment of VR dispersion in humans: do VCG-based dispersion measures reflect ischemia-induced dispersion changes? Another aim, related to the clinical issue of ischemia-induced arrhythmias and SCD, was to explore if there was a relation between changes in such dispersion measures and ventricular arrhythmias occurring during myocardial ischemia and reperfusion and, finally, with attempt to arrhythmia suppression. To this aim, we turned to an ischemia-reperfusion model in pigs.

## Methods

### Study subjects

**Figure 4:**
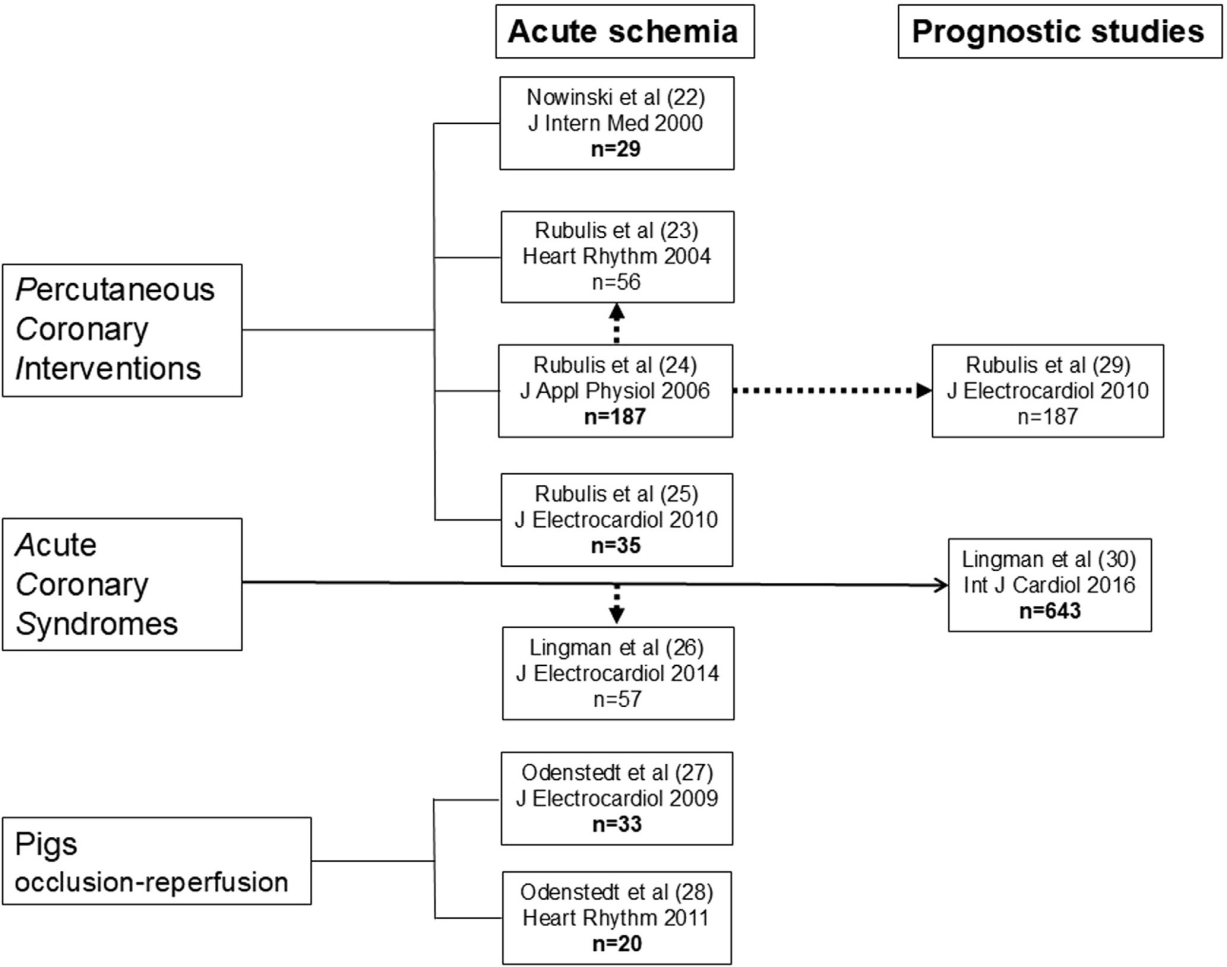
Overview of included studies and their relations. Numbers in bold font represent individual patients and pigs; some patients were included in more than one study as shown by arrows. Number within parenthesis next to author is reference number.

Figure 4 shows an overview of the 9 studies. Electrophysiological alterations were studied during acute ischemia induced by transient coronary occlusion in 251 patients with stable angina pectoris undergoing PCI.^22^, ^23^, ^24^, ^25^ In addition, electrophysiological alterations were studied from hospital admission in 57 patients with a first time acute anterior wall ST-segment elevation myocardial infarction (STEMI) and without documented prehospital ventricular arrhythmia.^26^ These 57 patients were selected from a cohort of 643 patients with acute coronary syndromes (ie, either STEMI, non-STEMI, or unstable angina pectoris). Furthermore, electrophysiological alterations and ventricular arrhythmias were studied during both ischemia and reperfusion in 53 pigs.^27^^,^^28^ Information about the studies on the effects of myocardial ischemia in humans and pigs is summarized in Table 1.

**Table 1:** Studies on electrophysiological effects of acute myocardial ischemia in humans and pigs using Frank vectorcardiography.

First author	Diagnosis	Culprit vessel (LAD/RCA/LCx)	Occlusion time (s)	Time from symptom onset (min)
Humans				
Nowinski^22^	Stable CAD, 29∗	10/10/10	171 ± 59	N/A
Rubulis^23^	Stable CAD, 56	28/17/11	37 ± 15	N/A
Rubulis^24^	Stable CAD, 187† with/without HT/LVH signs	89/50/29	37 ± 17	N/A
Rubulis^25^	Stable CAD, 35	14/7/14	128 ± 50	N/A
Lingman^26^	First STEMI, 57	57/—/—	N/A	75 (30–155)
Pigs				
Odenstedt^27^	Ischemia-reperfusion; 33	33/—/—	2700	N/A
Odenstedt^28^	ischemia-reperfusion; 20 (10 with/without spinal cord stimulation)	20/—/—	2700	N/A

Values are n, mean ± SD, or median (range).

CAD = coronary artery disease; HT = hypertension; LAD = left anterior descending artery; LCx = left circumflex artery; LVH = left ventricular hypertrophy; N/A = not applicable; RCA = right coronary artery; STEMI = ST-segment elevation myocardial infarction.

∗ 1 patient was included twice on separate occasions.

† 19 patients had more than 1 vessel treated and are not accounted for in the separate vessel groups.

Finally, the prognostic information of VCG-based measures was studied in 187 patients with stable angina pectoris after PCI and in the 643 patients referred to previously with a recent acute coronary syndrome.^29^^,^^30^

### VCG recording in humans and pigs

Continuous VCG monitoring was used. How this mode of monitoring during acute myocardial infarction came into being and led to a series of important studies in humans and pigs was covered by Sederholm.^31^ The present review focuses on electrophysiology and arrhythmogenicity, but it should be acknowledged that the initiative described by Sederholm was crucial for the development of the software platform on which our studies have been performed.

Electrode positions are shown in Figures 1A and 1B. The recording equipment was initially the MIDA (Myocardial Infarction Dynamic Analysis) 1000 or 1200 and later the Coronet system (Ortivus AB). The sampling rate was 500 Hz and filter settings were 0.03 to 170 Hz. The sampling periods varied between 10 seconds and 2 minutes depending on the aim of the study (ie, the signal-averaged QRST complex and the average loops were calculated from the dominant cardiac cycles during this time period). Methodological details are found in each publication.^22^, ^23^, ^24^, ^25^, ^26^, ^27^, ^28^, ^29^, ^30^

### The ST segment as the reference for myocardial ischemia

On the ECG, ST-segment deviations during ischemia are mainly thought to be due to changes in the action potential morphology and duration, and to the displacement of the T-Q segment (ie, the baseline).^32^^,^^33^ Myocardial cells within the ischemic zone have a higher (less negative) resting membrane potential and the plateau phase (phase 2) is shorter and downsloping due to accelerated repolarization caused by outflow of potassium ions compared with cells in nonischemic areas. During myocardial ischemia, the ST segment deviation is thus partly a reflection of VR dispersion caused by differences in action potential duration and morphology. ST segment changes on the ECG may, however, occur due to many physiological and pathophysiological factors.^34^

In the present context, we used the magnitude of the ST segment deviation from the isoelectric baseline as the “gold standard” for assessing the magnitude of myocardial ischemia.^31^ Furthermore, one of our studies in humans showed that the ST segment compared with other VCG-based measurers not only represents the earliest part of VR affected by myocardial ischemia, but also responded most accurately to the size of the myocardium at risk and was less dependent of location according to myocardial scintigraphy.^25^ The magnitude of the ST-segment deviation from the isoelectric baseline 60 ms after the J-point was therefore used for selecting the time points for data extraction.

### VCG-based measures and definitions

VCG-based measures and their definitions are presented in the Supplemental Material. The T vector loop is normally elliptical in a preferential plane, and the measures of T loop morphology, its roundness (Teigenvalue), and its planarity (Tavplan) are illustrated in Figure 2. For comparison with the terminology used by Badilini and colleagues,^13^ the 2 largest eigenvalues (ℷ_1_ and ℷ_2_) were used to calculate Teigenvalue, but we used the inverse relation between the two in our calculations, that is, (ℷ_1_/ℷ_2_)^2^, with all values ≥1 (1 for a perfect circle, which is most abnormal). The information of Teigenvalue comes close to that reflected by the so-called T-wave complexity.^35^ Tavplan was used to estimate the deviations of the loop from the preferential plane (ie, its “bulginess”). The set of VCG parameters was subsequently expanded to include Tamplitude and Tarea, reflecting global VR dispersion,^16^^,^^17^ and the VG (aka QRST_area_ or QRST integral or spatial VG), reflecting dispersion of action potential duration and morphology.^18^ In addition, the QRS-T angles were included when it became apparent that they provided significant prognostic information.^36^^,^^37^

### Changes in dispersion and arrhythmogenicity: The pig model

In brief, myocardial infarction was induced by occluding the left anterior descending coronary artery after the second diagonal branch by 45-minute balloon inflation. Reperfusion was then induced by deflating the balloon. The details of the model have been described elsewhere.^27^^,^^28^

### Prognostic value of VCG-based measures after PCI in patients with stable angina and after recent acute coronary syndromes

The cohort of 187 patients with stable angina was followed on average 8 years after PCI.^29^ In addition, 643 patients with a recent acute coronary event (myocardial infarction with/without ST-segment elevation and unstable angina) were followed 30 months.^30^ The evaluation of the latter cohort was performed according to the American Heart Association recommendations for evaluation of novel markers of cardiovascular risk.^38^ The additional prognostic value of several VCG-based parameters on top of 9 demographic and clinical variables was thus calculated using C-statistics and reclassification analysis with SCD and all cardiovascular deaths as 2 separate outcomes. The demographic and clinical variables were age, sex, diabetes, and previous stroke, and on admission left ventricular ejection fraction, estimated glomerular filtration rate, heart rate, systolic blood pressure <100 mm Hg, and Killip class >1.^30^

## Results

### T vector loop characteristics: Teigenvalue and Tavplan

**Figure 5:**
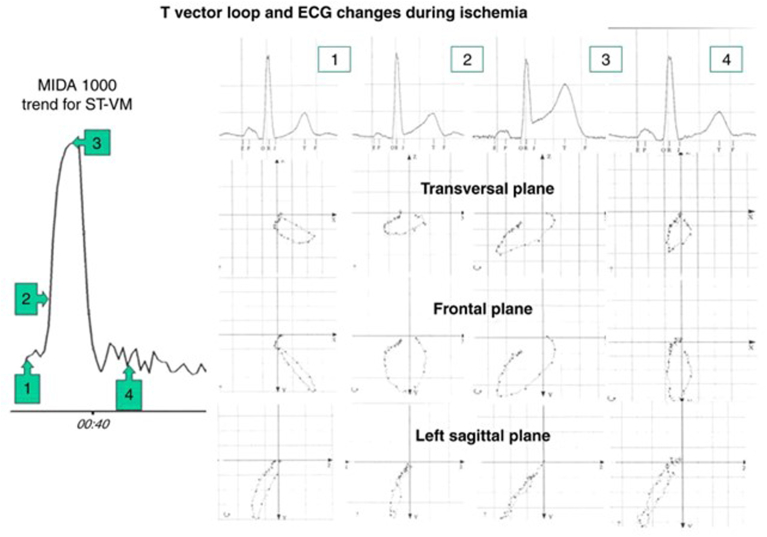
ST vector magnitude (ST-VM) and T loop changes during percutaneous coronary intervention to a right coronary artery. Number 1 denotes baseline; numbers 2 and 3 denote 15 and 60 seconds after start of the last (fourth) balloon occlusion, respectively; and number 4 denotes 20 seconds after balloon deflation. During occlusion, there is a concurrent increase in ST-VM and an increasingly rounder and bulgier T vector loop; as a consequence, Teigenvalue decreases and Tavplan increases. Reproduced with permission from Rubulis A. T-vector and T-loop morphology analysis of ventricular repolarization in ischemic heart disease. PhD thesis, Karolinska Institutet, Stockholm, Sweden; 2007, and Rubulis and colleagues (Elsevier license 5880220780767).^25^

As stated previously, the T vector loop has a preferential plane in space that varies individually. It is normally elliptical and with limited bulginess. During angioplasty, the T vector loop became more circular, with a 30% to 60% decrease in Teigenvalue, and bulgier, with a 40% to 50% increase in Tavplan (Figure 5).^22^, ^23^, ^24^, ^25^ In patients with vs without ECG signs of left ventricular hypertrophy, both parameters were more abnormal already at baseline, and this abnormality was aggravated by ischemia.^24^ During the acute phase of a first-time anterior STEMI, Teigenvalue was 50% lower and Tavplan was 90% higher compared with the subacute phase, in line with results during PCI. Acute myocardial ischemia thus significantly alters T vector loop morphology in the direction of a more circular (less elongated) and bulgier (less planar) loop.

### Tarea, Tamplitude, and VG

Tarea increased by 33% to 87% during PCI, most in patients with hypertension, and was 188% larger during the acute vs subacute phase of an anterior STEMI (Table 2).^23^, ^24^, ^25^, ^26^ Neither Tamplitude nor VG was analyzed in the PCI studies, but both were significantly altered during the acute vs subacute phase of an anterior STEMI: Tamplitude was 14% lower and VG was 159% larger.^26^ These 3 VCG-based dispersion parameters thus changed significantly during acute myocardial ischemia.

**Table 2:** Electrophysiological effects of acute ischemia in humans studied with Frank vectorcardiography.

First author	Tarea	Tamplitude	VG	QRS-T angle	Tavplan	Teigenvalue
Nowinski^22^	+33%∗	N/A	N/A	N/A	+52%∗∗	–59%∗
Rubulis^23^	+50%∗∗∗	N/A	N/A	±0 (peak)	∼+40%∗∗∗	∼–30%∗∗∗
Rubulis^24^		N/A	N/A			
Controls	+40%∗∗			+22% (peak)∗∗∗	+50%∗∗	–63%∗∗
HT	+87%∗∗			+4% (peak)	+50%∗∗∗	–8%
LVH	+61%∗∗			±0% (peak)	+14%	–25%
Rubulis^25^	+33%∗∗	N/A	N/A	+9% (peak)	+104%∗∗	–43%∗
Lingman^26^	+188%∗∗	–14%∗∗	+159%∗∗	–12% (peak) –26% (mean)∗∗	+90%∗∗	+5%

HT = hypertension; LVH = left ventricular hypertrophy; N/A = not available; VG = ventricular gradient.

∗ *P* < .05.

∗∗ *P* < .001.

∗∗∗ *P* < .01.

### Observations from the pig occlusion-reperfusion model

The VCG alterations were mostly similar in the pig model compared with those in humans. The T vector loop thus became more circular and bulgier, and the Tarea increased (but the change in the QRS-T angle was opposite, as expected for anatomical reasons).^27^ Although the native coronary artery system is similar in pigs and humans (noncollateralized), the Purkinje network is not subendocardial as in humans but penetrates the ventricular wall. Consequently, the QRS-T angle is normally very wide in pigs before intervention. Furthermore, when comparing pigs with (n = 16) vs without (n = 17) ventricular fibrillation (VF), a bulgier T vector loop and increased heart rate were observed preceding the VF episode. In a subsequent study, the electrophysiological and antiarrhythmic effects of spinal cord stimulation were compared in 10 pigs with vs 10 pigs without this intervention. Spinal cord stimulation reduced the number of episodes of sustained and nonsustained ventricular tachycardia and reduced the VCG changes but had no effect on VF (n = 6), myocardium at risk, or infarct size.^28^^,^^29^

The VCG results obtained in the pig model were thus very similar to those in our human studies. Furthermore, and more crucially, aggravated alteration in T vector loop morphology was associated with the appearance of VF, and reduction of several VCG parameters reflecting VR dispersion was associated with a reduction in ventricular arrhythmias. A link between changes in VCG-based dispersion parameters and ventricular arrhythmogenicity and an antiarrhythmic intervention was thus established.

### VCG and the prognosis after PCI in patients with stable angina

In multivariate analysis, cardiovascular deaths were predicted by signs of left ventricular hypertrophy, left ventricular ejection fraction, QRS duration, and the peak QRS-T angle (>101°), corroborating the results of previous studies. A new observation was that Tavplan (>0.71 μV) predicted a new myocardial infarction.^29^

### Additional prognostic value after recent acute coronary syndromes

The mean and peak QRS-T angles had independent predictive value for both SCD and all cardiac deaths at 30 months follow-up after an acute coronary event.^30^ Furthermore, reclassification analysis proved their additional prognostic value. For SCD, the net reclassification improvements were 46% and 45% for the mean and peak QRS-T angles, respectively. The so-called relative integrated discriminative improvement was 16% and 13% vs the average ∼11% of the 9 demographic and clinical risk factors. The resulting area under the receiver-operating characteristic curves were approximately 0.87 for SCD and 0.88 for all cardiac deaths. For SCD, the logarithmic value of T_elevation_ (ie, the caudocranial direction of the T vector) also significantly improved classification of both events and nonevents correctly, with an relative integrated discriminative improvement of 20%. In addition, both QRS_amplitude_ and the logarithmic value of QRS_area_ seemed to add prognostic value but much less than the QRS-T angles. In contrast, the VG was not an independent predictor for any cardiac death in the adjusted analysis.

## Discussion

In a series of studies in humans and pigs performed during a period of approximately 2 decades, we tested how VCG-based dispersion parameters changed during myocardial ischemia, which is known to cause regional differences in action potential duration and morphology. In the pig coronary occlusion-reperfusion model, we compared the VCG responses in pigs with vs without VF with those in humans, and subsequently the effects of arrhythmia suppression by spinal cord stimulation. The main findings were that (1) it was validated that changes in VCG-based dispersion parameters reflected ischemia-induced action potential heterogeneities during coronary occlusion/myocardial infarction, similarly in humans and pigs; (2) accentuated VCG-based dispersion parameters and increased heart rate preceded VF in pigs, and spinal cord stimulation reduced the magnitude of VR dispersion and sustained and nonsustained ventricular tachycardia but not VF; (3) after acute coronary syndromes, increased peak and mean QRS-T angles added predictive value regarding subsequent cardiac death including SCD on top of 9 demographic and clinical risk factors, while VCG parameters reflecting changes in dispersion during acute ischemia did not; and (4) the T vector loop was more abnormal (more circular and bulgier) in patients with amplitude signs of left ventricular hypertrophy on ECG and became more abnormal during acute myocardial ischemia (ie, T vector loop morphology changes have pathophysiological correlates). Taken together, our observations not only corroborate the results and conclusions reached by Badilini and colleagues^13^ and Zabel and colleagues,^39^ but also add information about the link between noninvasive VCG-based electrophysiological parameters with myocardial ischemia, arrhythmogenicity, and antiarrhythmic intervention.

Acute myocardial ischemia is a major cause of potentially life-threatening ventricular arrhythmias and a condition with altered/increased ventricular dispersion. Routine PCI induces myocardial ischemia and offers an opportunity to study and validate alterations in VCG-based parameters, especially at longer balloon inflation times (as used in the past). The pathophysiological background and its effects on the cellular and organ level and ECG is well established.^5^, ^6^, ^7^^,^^32^^,^^33^ There are clear differences in action potential duration and morphology between myocytes within the ischemic zone and myocytes in nonischemic areas. The dispersion caused by these differences can set up the stage for arrhythmias.

For repolarization dispersion to be arrhythmogenic, spatial differences between adjacent areas such as in myocardial ischemia due to coronary occlusion is one but not the only mechanism. Postrepolarization refractoriness is another factor. Additionally, VR duration and dispersion is heart rate dependent due to nonuniform recovery of excitability on the cellular level in different parts of the ventricles and many arrhythmias occur in a context of changing heart rate.^40^, ^41^, ^42^, ^43^, ^44^, ^45^, ^46^, ^47^, ^48^ Temporal heterogeneity of rate adaptation as well as the timing and the site of origin of the triggering extrasystole are also important arrhythmogenic factors.^49^^,^^50^ Alterations and differences in the timing of the electrophysiological adaptation to changes in heart rate might thus be arrhythmogenic even in the absence of signs of local/regional differences in action potential duration and morphology such as those observed in myocardial ischemia. Spatiotemporal dispersion of repolarization is both complex and dynamic.

### T vector loop morphology and QT dispersion revisited

Kors and colleagues^14^ stated in the first sentences of the Discussion in their influential publication from 1999,

We argued on physical grounds that a commonly suggested explanation for the mechanism underlying QTD (*QT dispersion; au comment*), i.e., local differences in action potential durations, does not hold. Instead, we propose an alternative explanation that relates QTD to T-wave morphology. We showed that ECGs with narrow, tall T loops have relatively small QTD values, whereas wide, small loops have the largest QTDs.

Against the background of the results of our studies, we would add to this statement and on pathophysiological grounds argue as follows: during acute myocardial ischemia, experimental as well as clinical differences in action potential duration and morphology between ischemic and nonischemic tissue not only cause ST-segment deviations, but also changes in the T vector loop morphology. The loop becomes less elongated (more circular) and bulgier,^22^, ^23^, ^24^, ^25^ and during simultaneous recordings of VCG and 12-lead scalar ECG there is a correlation between these T vector loop morphology changes and increased QT dispersion.^22^ In addition, in patients with stable angina and hypertension with vs without ECG signs suggesting myocardial hypertrophy, the T vector loop morphology before coronary occlusion is already less elongated and bulgier and these abnormalities become more accentuated during ischemia.^24^ Furthermore, and according to another study from our group applying the same VCG methodology, congenital LQT1 and LQT2 are associated with a more circular and bulgier T vector loop than healthy controls.^51^

We do, however, not argue for a renaissance of QT dispersion assessment for noninvasive evaluation of repolarization dispersion but rather argue for the value of VCG, as suggested by Badilini and colleagues almost 30 years ago.^13^ QT dispersion, the interlead differences between the QT intervals, has several limitations as pointed out elsewhere, one being that the overall VR duration is not reflected by the QT interval in any selected ECG lead.^52^ The QRST complex in each ECG lead represents the projection of the QRS and T vector loops on that lead and is affected by the orientation of the loops in relation to the specific lead. Using VCG according to Frank this is not an issue, a clear advantage. In the 3-dimensional or global QRST complex derived from the X, Y, and Z leads, there is only 1 QRS onset and 1 T-wave end (Figure 1D). The QT interval is therefore unaffected by the loop axes orientation. Consequently, VCG-based measurement of the QTc interval—the global QTc—was more accurate than both manual and automatic QTc analyses on scalar ECG for diagnosing long QT syndrome.^53^

### Methodological aspects and limitations

Many important studies on cardiac electrophysiology and arrhythmias during myocardial ischemia have not been mentioned in this report. The reason is that the aim never was to review and cover all aspects of this significant and vast field, but merely to share the experience from our studies applying VCG according to Frank with a consistent methodology for the study of VR dispersion and its changes and put them into a common context. The limitations of each individual study are accounted for in the original publications on which this review is based.

Apart from the first study,^22^ scalar ECG was not recorded simultaneously with VCG, which obviously would have provided additional information and possibilities for comparisons. Scalar ECG can be used to calculate approximations of VCG and the other way around. However, we would like to emphasize that VCG offers advantages vs scalar ECG for several purposes, not only regarding the QT interval for diagnosing LQTS, the assessment of VR dispersion instead of QT dispersion, and in the prediction of responders to cardiac resynchronization therapy and adjustment of device programming.^51^^,^^54^, ^55^, ^56^, ^57^ The anatomical representation becomes more accurate due to the electrodes positioned on the back and neck. This is especially important for detecting alterations in the transversal plane (ie, azimuth) and within or toward the sagittal plane. Such changes are common after ablation of overt left lateral accessory pathways in the Wolff-Parkinson-White syndorme and in abnormal QRS-T angles, the latter being one explanation for why VCG is the “gold standard” for the assessment of QRS-T angles.^19^^,^^58^ And abnormal QRS-T angles are strong risk predictors for cardiac death including SCD.^21^

While the QRS-T angles are prognostically important, they are not sensitive to acute ischemia. Tavplan, on the other hand, is very sensitive to acute myocardial ischemia and had an independent prognostic value for predicting subsequent myocardial infarction after PCI in patients with stable angina.^25^ This observation obviously needs confirmation but does not seem unreasonable. Mechanistically, Tavplan expresses a variation of the T vector direction and/or amplitude in relation to the preferential plane of the loop. It may, at least tentatively, be regarded as a variant of T-wave alternans on the microvolt level. T-wave alternans has attracted much attention during the last decades especially for prediction of ventricular arrhythmias and SCD in patients with coronary artery disease, long QT syndromes, and other cardiac and noncardiac diseases (almost 2000 entries in PubMed the last 30 years), and the relation between myocardial infarction, ventricular arrhythmia, and SCD is unquestionable.

During PCI, ventricular arrhythmias are rare, and the relation between VR dispersion and its changes and arrhythmogenicity can therefore not be studied. Furthermore, in the study of patients admitted with an ongoing acute anterior STEMI, prehospital ventricular arrhythmia was an exclusion criterion because we wanted to study the “natural course” of VR during ischemia-reperfusion in humans after hospital admission.^26^ In contrast to PCI in humans, ventricular arrhythmias are rather frequent in the pig occlusion-reperfusion model. Studies of the relation between repolarization dispersion and its changes and arrhythmias were therefore possible in the pig model.

## Conclusion

Noninvasive electrophysiological parameters based on VCG were validated for studying changes in VR dispersion during acute myocardial ischemia and reperfusion in humans and pigs. They included Tamplitude, Tarea, and the VG, corroborating previous studies, but also T vector loop morphology parameters Teigenvalue and Tavplan, reflecting the roundness and bulginess of the loop. A relation between changes in such parameters and the development of ventricular arrhythmias and their suppression by spinal cord stimulation was shown in the pig model. For prognostic purposes after acute coronary syndromes, the parameters that were most sensitive to acute ischemia were not so useful, and instead the peak and mean QRST angles were superior.
