# Supplementary material for: Ischemia induced repolarization dispersion changes and ventricular arrhythmia: Validation of Frank vectorcardiography parameters: A review
Source: Heart Rhythm O2. 2025 Feb 17;6(5):709–19. doi: 10.1016/j.hroo.2025.02.005 (PMC12147597; doi:10.1016/j.hroo.2025.02.005)
Supplement: Supplementary Material [file mmc1.docx]

**Supplement**

Glossary and definitions

**Mean QRS-T angle** The angle between the QRSarea vector and the Tarea vector [also

referred to as the QRS-T area angle (0° to 180°)]

**Peak QRS-T angle** The angle between the maximum QRS and T vectors in space and inscribed in the QRS- and T-vector loops, also referred to as the QRS-T angle (0° to 180°)

**QRSarea**  The spatial area between the baseline and the curve formed by the moving vector during the QJ interval, QRSarea = (QRSx^2^ + QRSy^2^ + QRSz^2^)^1/2^ [µVs]. Also, a vector and part of ventricular gradient.

**QT** The interval measured in the 3-dimensional QRST complex from QRS onset to Tend; also, the global QT interval [ms]

**QTc** Heart rate corrected QT interval normalized to a heart rate of 60 bpm [ms]

**Tamplitude** The maximum T amplitude in space (maximum vector inscribed

in the T-vector loop) [mV]

**Tarea**  The spatial area under the curve formed by the moving heart vector during the interval from QRS offset (J-point) to end of T; Tarea = (Tx^2^ + Ty^2^ + Tz^2^)^1/2^ [µVs]. Also, a vector and part of ventricular gradient.

**Tavplan** The bulginess of the T-vector loop computed as the mean absolute distance between the periphery of the T loop and the preferential plane; reflects the loop’s lack of planarity, higher values represent a more abnormal T loop [μV]

**Teigenvalue** The roundness of the T-vector loop (unit less), computed as the squared quotient between the two largest perpendicular axes (eigenvalues) of the T loop in the preferential plane (d1/d2)^2^ (where d1 ≥ d2); high value in healthy hearts and never more abnormal than 1.0 for a circle

**VCG** Vectorcardiogram applying an orthogonal lead system

(XYZ) according to Frank [in the literature sometimes estimated from the 12-lead ECG]

**VG**  Ventricular gradient or QRST area integral is the spatial area under the curve formed by the moving heart vector during the QT interval; also, the vector sum of the QRSarea and Tarea vectors (aka spatial ventricular gradient; SVG) [µVs]
